# Steroid Anti-Inflammatory Effects Did Not Improve Organ Quality in Brain-Dead Rats

**DOI:** 10.1155/2015/207534

**Published:** 2015-05-19

**Authors:** Rolando A. Rebolledo, Bo Liu, Mohammed Z. Akhtar, Petra J. Ottens, Jian-ning Zhang, Rutger J. Ploeg, Henri G. D. Leuvenink

**Affiliations:** ^1^Department of Surgery, University Medical Center Groningen, 9713 GZ Groningen, Netherlands; ^2^Department of Surgery, Faculty of Medicine, University of Chile, 8380453 Santiago, Chile; ^3^Department of Neurosurgery, Tianjin Medical University General Hospital, Tianjin 300052, China; ^4^Nuffield Department of Surgical Sciences, University of Oxford, Oxford OX3 9DU, UK

## Abstract

Effect of glucocorticoid administration on improving the outcomes of kidney and liver allografts has not been clearly elucidated. This study investigated the effect of prednisolone administration after onset of brain death (BD) on kidney and liver in a controlled rat model of BD. BD was induced in rats by inflating an epidurally placed balloon catheter. Animals were treated with saline or prednisolone (5, 12.5, or 22.5 mg/kg) one hour after the onset of BD. After 4 hours of BD, experiments were terminated and serum and tissues were collected. Tissue gene and protein expression were measured for markers of inflammation, apoptosis, and cellular stress response markers. Prednisolone caused a reduction of plasma levels of IL-6, while the tissue expression of *IL-6, IL-1β,* and *MCP-1* in both kidney and liver were also reduced. Creatinine plasma levels, complement (*C3*) expression, *HSP-70, HO-1, Bcl2/BAX* ratio, and PMN influx did not significantly change in kidney nor liver. Plasma AST and LDH levels were increased in the prednisolone treated group. Our results demonstrate prednisolone can has an anti-inflammatory effect mediated through reducing serum circulating cytokines. However, this anti-inflammatory effect does not translate into improved kidney function and indeed was associated with increased liver injury markers.

## 1. Introduction

The shortage of organs for transplantation is one of the most important problems facing the transplant community today. Identifying how injury occurs in donor organs and how this injury can be ameliorated will result in increased numbers of suitable organs for transplantation.

To address this, a number of different approaches have been described for the management and optimisation of BD organ donors. There is general consensus about the importance of maintaining hemodynamic stability. As a consequence donor care bundle pathways have been developed and include targets for mean arterial pressures and cardiac output amongst other physiological parameters [1].

The role of hormonal or anti-inflammatory treatments remains controversial however despite some promising experimental evidence [2, 3]. Many donor management protocols usually include steroids, but this indication is based on poor quality evidence and is subject to considerable debate in the literature [4–9]. The distinguishing effects of glucocorticoid administration on the kidney and liver have not been fully elucidated.

Brain death causes a complex disturbance of normal homeostatic systems resulting in hemodynamic instability [10–13], hormonal impairment [14–16], and inflammation [17]. Brain death is the result of significant cerebral ischemia and intracranial hypertension resulting in parasympathetic activity followed by severe vasoconstriction due to an overriding sympathetic response by endogenous catecholamines. This catecholamine activity is also called the* autonomic* or* catecholamine storm* and is part of the Cushing reflex, a physiological response to maintain cerebral perfusion. Because of the progressive paralysis of the spinal cord and the loss of vasomotor tone, hemodynamic instability characterizes this period [18–20].

In addition to hemodynamic instability, hormonal secretion is altered. Pituitary function is affected as ACTH secretion is initially increased, resulting in a transient rise in cortisol levels during brain death; this then diminishes however, progressing to below baseline levels [3]. A reduction in thyroid stimulating hormone (TSH) also occurs.

During brain death, a systemic inflammatory state is characterized by circulating cytokines, including interleukin-6 (IL-6), interleukin-10 (IL-10), Tumor Necrosis Factor-alpha (TNF-*α*), Transforming Growth Factor-beta (TGF-*β*), and also monocyte chemotactic protein 1 (MCP-1); it is widely reported [21–23]. This inflammatory environment permits an influx of inflammatory cells into organs including the kidneys, liver, and lung, leading to a local inflammatory response culminating in cellular apoptosis [24, 25]. The origin of this inflammatory status is not well understood but may include the release of cerebral inflammatory cytokines which cross a disrupted blood brain barrier, complement activation [26, 27], and increased intestinal permeability [28].

In brain death glucocorticoid administration could have several beneficial effects including anti-inflammatory properties and the ability to augment chromaffin cells production of endogenous adrenaline [29]. The anti-inflammatory effects of steroids result from the pleiotropic interaction with the glucocorticoid receptor. The cortisol-glucocorticoid receptor complex can act through genomic and nongenomic downstream signalling pathways within the cell. This involves the dissociation of heat shock proteins and the interaction with membrane-associated receptors and second messengers and activation of transcription factors such as nuclear factor kappa-light-chain-enhancer of activated B cells (NF-*κ*B) [30].

We investigated whether the quality of liver and kidney grafts could be improved by prednisolone treatment after brain death induction in a rodent model.

We hypothesised that prednisolone treatment administered after brain death induction could reduce the inflammatory response in brain-dead rats and improve kidney function but not liver cellular injury based on our previous work [31].

## 2. Methods

### 2.1. Animal Brain Death Model

All animals received care in compliance with the guidelines of the Institutional Animal Care and Use Committee-Rijksuniversiteit Groningen (IACUC-RUG) according to Experiments on Animals Act (1996) issued by the Ministry of Public Health, Welfare and Sports of Netherlands. The IACUC-RUG approved this study and all animal care actions. Male adult Fisher F344 rats (250–300 g) were used. Brain death (BD) was induced as described previously [32]. In brief, the procedure was as follows: animals were anesthetized using isoflurane with O_2_. Cannula was inserted in the femoral artery and vein for continuous mean arterial pressure monitoring and administration of fluids. Animals were intubated via a tracheostomy and ventilated throughout the experiment. Through a frontolateral hole in the skull, a number 4 Fogarty catheter (Edwards Lifesciences Co, Irvine, CA) was placed epidurally and slowly inflated (16 *μ*L/min) with saline using a syringe pump (Terufusion, Termo Co, Tokyo, Japan). During balloon inflation, a hypotensive period of about 15 minutes occurred followed by a sudden increase in blood pressure. When the blood pressure returned to its basal level (80 mmHg) during the increasing peak, inflation of the balloon was stopped and anesthesia was withdrawn. The balloon was kept inflated until the end of the experiment. Brain death was confirmed 30 min after anesthesia was terminated by the absence of corneal and pupillary reflexes. Mean arterial blood pressure was maintained above 80 mmHg throughout the BD period using HAES 10% (Fresenius Kabi AG, Bad Homburg, Germany) in bolus of 0.5 mL with a maximum infusion rate of 1 mL/hr. If HAES had insufficient effect on blood pressure, noradrenaline (NA) 0.01 mg/mL infusion was administered. A homeothermic blanket control system was used throughout the BD maintenance period. Four hours after determination of BD, a laparotomy was performed and blood was collected from the aorta. Organs were subsequently flushed with 0.9% cold saline and snap frozen in liquid nitrogen. Blood was centrifuged for 10 min at 960 g and plasma collected and stored at −80°C.

Rats were randomly divided, each group consisting of six to eight animals. Prednisolone or saline was administered intravenously, one hour after BD induction. Prednisolone dosage was chosen based on previous experiments.

The following experimental groups can be distinguished:

- Brain-dead animals receiving saline (*n* = 7).
- Brain-dead animals receiving 22.5 mg/Kg prednisolone (*n* = 7).
- Brain-dead animals receiving 12.5 mg/Kg prednisolone (*n* = 8).
- Brain-dead animals receiving 5 mg/Kg prednisolone (*n* = 6).

### 2.2. Plasma Determinations

At the Laboratory Center of the University Medical Center Groningen (Mega, Merck), the following measurements were determined in a routine fashion: creatinine in plasma by the Jaffe method [33] and alanine aminotransferase and aspartate aminotransferase enzyme activity in plasma [34].

The level of IL-6 in the plasma was determined by a rat enzyme-linked immunosorbent assay (IL-6 ELISA) kit (R&D Systems Europe Ltd. Abingdon, Oxon OX14 3NB, UK), according to the manufacturer's instructions. All samples were analyzed in duplicate and read at 450 nm.

### 2.3. RNA Isolation and cDNA Synthesis

Total RNA was isolated from whole kidneys and liver sections by using TRIzol (Life Technologies, Gaithersburg, MD). RNA samples were verified for absence of genomic DNA contamination by performing RT-PCR reactions in which the addition of reverse transcriptase was omitted, using glyceraldehyde 3-phosphate dehydrogenase (GAPDH) primers. For cDNA synthesis, 1 *μ*L T11VN Oligo-dT (0.5 *μ*g/*μ*L) and 1 *μ*g mRNA were incubated for 10 min at 70°C and cooled directly after that. cDNA was synthesized by adding a mixture containing 0.5 *μ*L RnaseOUT ribonuclease inhibitor (Invitrogen, Carlsbad, USA), 0.5 *μ*L RNase water (Promega), 4 *μ*L 5x first strand buffer (Invitrogen), 2 *μ*L DTT (Invitrogen), 1 *μ*L dNTPOs, and 1 *μ*L M-MLV reverse transcriptase (Invitrogen, 200 U). The mixture was held at 37°C for 50 min. Next, reverse transcriptase was inactivated by incubating the mixture for 15 min at 70°C. Samples were stored at −20°C.

### 2.4. Real-Time PCR

Fragments of several genes were amplified with the primer sets outlined in Table 1. Pooled cDNA obtained from brain-dead rats was used as internal references. Gene expression was normalized with the mean of *β*-actin mRNA content. Real-Time PCR was carried out in reaction volumes of 15 *μ*L containing 10 *μ*L of SYBR Green mastermix (Applied biosystems, Foster City, USA), 0.4 *μ*L of each primer (50 *μ*M), 4.2 *μ*L of nuclease free water, and 10 ng of cDNA. All samples were analyzed in triplicate. Thermal cycling was performed on the TaqMan Applied Biosystems 7900HT Real-Time PCR System with a hot start for 2 min at 50°C followed by 10 min 95°C. Second stage was started with 15 s at 95°C (denaturation step) and 60 s at 60°C (annealing step and DNA synthesis). The latter stage was repeated 40 times. Stage 3 was included to detect formation of primer dimers (melting curve) and begins with 15 s at 95°C followed by 60 s at 60°C and 15 s at 95°C. Primers were designed with Primer Express software (Applied Biosystems) and primer efficiencies were tested by a standard curve for the primer pair resulting from the amplification of serially diluted cDNA samples (10 ng, 5 ng, 2.5 ng, 1.25 ng, and 0.625 ng) obtained from brain-dead rats. PCR efficiency was found to be 1.8 < *ϵ* < 2.0. Real-Time PCR products were checked for product specificity on a 1.5% agarose gel. Results were expressed as 2^−ΔΔCT^ (CT: Threshold Cycle).

### 2.5. Immunohistochemistry

To detect polymorphonuclear cells (PMN) in kidney and liver, immunohistochemistry was performed on 5 *μ*m tissue cryosections. Sections were fixated for 10 min using acetone. Next, sections were stained with HIS-48 mAb (supernatant, two times diluted) using an indirect immunoperoxidase technique. Endogenous peroxidase was blocked using H_2_O_2_ 0.01% in phosphate-buffered saline for 30 min. After thorough washing, sections were incubated with horseradish peroxidase-conjugated rabbit anti-mouse IgG as a secondary antibody for 30 min, followed by goat anti-rabbit IgG as a tertiary antibody for 30 min (both from Dako, Glostrup, Denmark). The reaction was developed using 9-amino-ethylcarbazole as chromogen and H_2_O_2_ as substrate. Sections were counterstained using Mayer hematoxylin solution (Merck, Darmstadt, Germany). Negative antibody controls were performed. Localization of immunohistochemical staining was assessed by light microscopy. For each tissue sample, positive cells were counted in 10 microscopic random fields of the tissue at 40x magnification. Results were presented as number of positive cells per glomerulus in the kidney and number of positive cells per area (*μ*m^2^) in the liver.

### 2.6. Statistical Analysis

For statistical analysis between the four experimental groups, the Kruskal-Wallis test was performed, followed by Dunn's posttests for comparison between groups, with *p* < 0.05 regarded as significant. Results are presented as mean ± SD (standard deviation). Statistical analyses were performed using Prism 5.0. GraphPad.

## 3. Results

**Figure 1:**
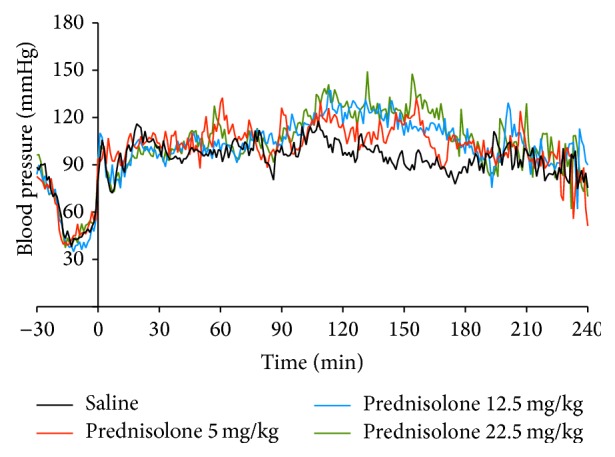
Blood pressure profile. The record started with the brain death induction; we considered time 0 as the end of brain death induction and the starting of brain death period.

Induction of brain death showed a consistent and uniform pattern in blood pressure with a mean induction time of 32,5 ± 2,5 minutes which is similar to our previous publications [31]. After brain death induction all animals were kept with a mean arterial pressure of above 80 mmHg during the remainder of the experiment (Figure 1). No significant difference was found in the requirement of HAES 10% (*p* = 0.44). The volume of noradrenaline required to keep blood pressure above 80 mmHg was significantly higher in the saline group (*p* = 0.04, Table 2).

**Figure 2:**
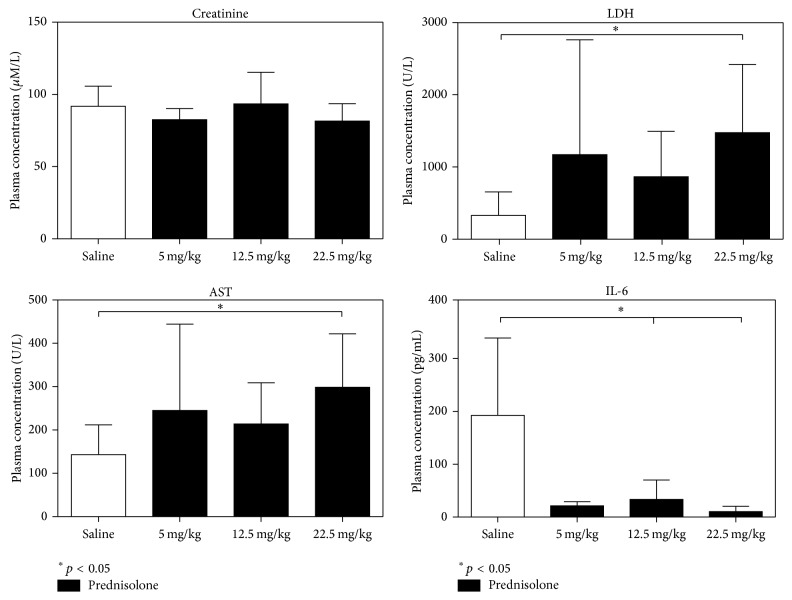
Plasma levels of kidney function marker (creatinine), liver injury markers (AST, LDH, and ALT), and interleukin-6 (IL-6).

No significant difference was found in creatinine levels between BD saline condition and prednisolone groups. An increase of aspartate aminotransferase (AST) and lactate dehydrogenase (LDH) plasma levels was associated with the groups treated with the higher prednisolone dose compared with saline treated group (AST: saline, 143.1 ± 68.59 U/L and 22.5 mg/Kg prednisolone group, 298.7 ± 123.1; LDH: saline, 331.7 ± 324.0 U/L and 22.5 mg/Kg prednisolone group, 1474 ± 944.1 U/L). A decrease of IL-6 plasma levels was found in all prednisolone groups compared with saline treated group (IL-6: saline, 192 ± 146.3 pg/mL; 5 mg/Kg prednisolone group, 21.33 ± 7.68 pg/mL; 12.5 mg/Kg prednisolone group, 33.63 ± 36.68 pg/mL; and 22.5 mg/Kg prednisolone group, 10.57 ± 9.79 pg/mL) (Figure 2).

**Figure 3:**
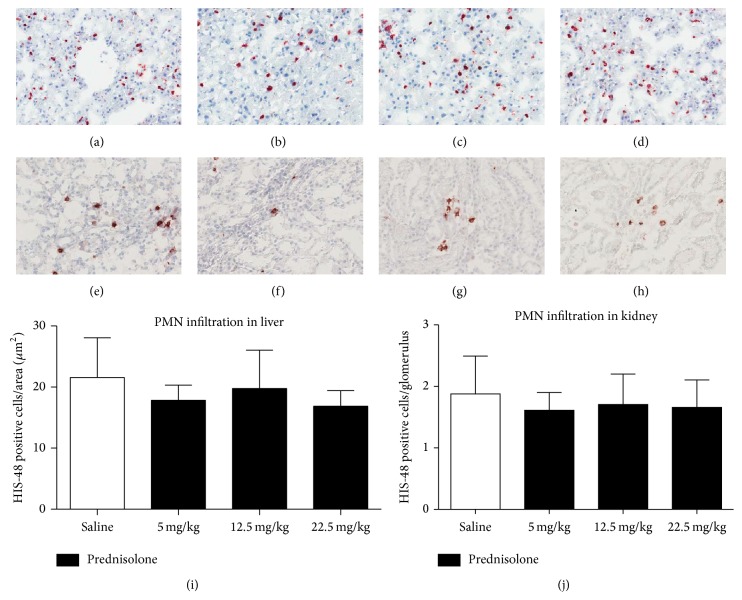
PMN infiltration quantification and staining. (a) Liver tissue from a BD animal treated with saline. (b) Liver tissue from a BD animal treated with 22.5 mg/Kg of prednisolone. (c) Liver tissue from a BD animal treated with 12.5 mg/Kg of prednisolone. (d) Liver tissue from a BD animal treated with 5 mg/Kg of prednisolone. (e) Kidney tissue from a BD animal treated with saline. (f) Kidney tissue from a BD animal treated with 22.5 mg/Kg of prednisolone. (g) Kidney tissue from a BD animal treated with 12.5 mg/Kg of prednisolone. (h) Kidney tissue from a BD animal treated with 5 mg/Kg of prednisolone. (i) Quantification of liver samples. (j) Quantification of kidney samples.

**Figure 4:**
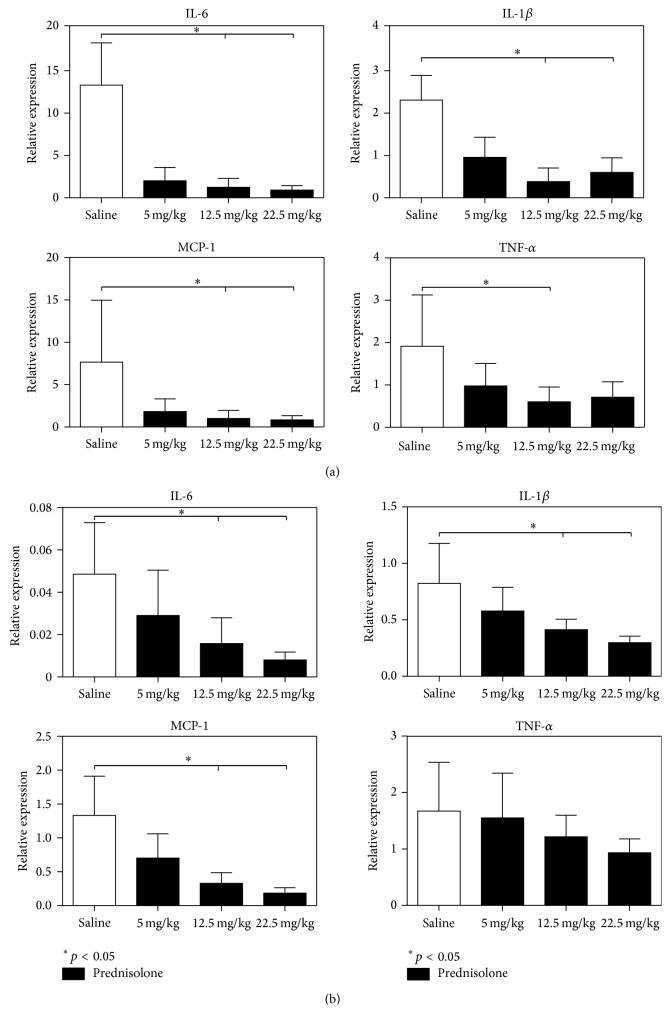
Relative expression of inflammatory genes. (a) Kidney and (b) liver.

In order to assess the anti-inflammatory effects of the prednisolone treatment PMN influx was studied as well as the expression of inflammatory genes in livers and kidney. No significant difference was found in PMN influx to the kidney nor liver due to prednisolone treatment (Figure 3). A decrease in* TNF-α, IL-6, IL-1β*, and* MCP-1* expression was found in kidneys of rodents treated with prednisolone compared with the control group. In the liver* IL-6, IL-1β*, and* MCP-1* were downregulated in the prednisolone groups but the expression of* TNF-α* was not modified by this treatment (Figure 4).

**Figure 5:**
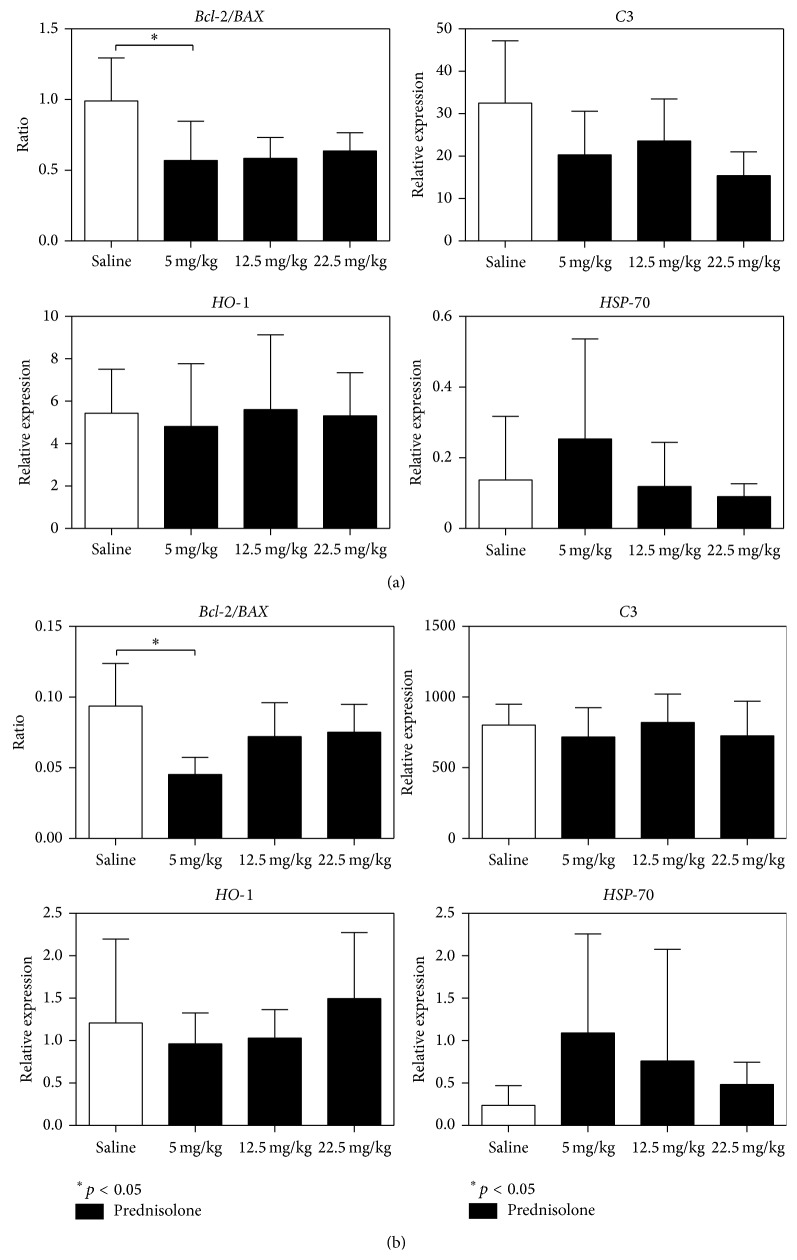
Relative expression of* C3* complement, heme oxygenase-1 (*HO-1*), and heat shock protein 70 (*HSP-70*). Ratio of the relative expression of* Bcl-2/BAX*. (a) Kidney and (b) liver.

To assess complement activation, which has previously been shown to be associated with brain death induced organ injury, we studied the expression of the complement component 3 (*C3*) in kidney and liver [35]. The relative* C3* expression did not change due to prednisolone treatment in both organs (Figure 5).

We studied the apoptotic pathway by B-cell lymphoma 2 (*Bcl-2*)/Bcl-2 associated X protein (*BAX*) ratio. No change was found in the 22.5 mg/Kg nor 12.5 mg/Kg prednisolone treated groups when compared with the saline treated group, while a decrease in the* Bcl-2/BAX* ratio was found using the lower dose of prednisolone in liver and kidney (*Bcl-2/BAX* kidney: saline, 0.99 ± 0.30 and 5 mg/Kg prednisolone group;* Bcl-2/BAX* liver: saline, 0.09 ± 0.03 and 5 mg/Kg prednisolone group, 0.04 ± 0.01) (Figure 5).

We studied the effect of the interventions on the relative expression of two cytoprotective genes, heme oxygenase 1 (*HO-1*) and* HSP-70*. Both the expressions of* HO-1* and* HSP-70* were not modified in liver or kidney by the prednisolone treatment (Figure 5).

## 4. Discussion

Disparity exists in the literature with regard to the beneficial effects of steroids administration to the donor on the outcomes of solid organ transplantation. Two recent systematic reviews of Dupuis et al. and Rech et al. [4, 36] concluded that evidence supporting the routine use of steroids in the management of organ donors is conflicting. The lack of knowledge about the effect of steroids on specific organs such as the kidney and liver with regard to donation was the stimulus to design and perform this study. We hypothesized that prednisolone administration would reduce the donor inflammatory response and improve the quality of both kidney and liver allografts. Our initial observation concerned improvements in the hemodynamic status of the brain-dead rodents treated with prednisolone.

We have demonstrated that prednisolone improves the hemodynamic status significantly reducing the noradrenaline requirements. Dhar et al. [37] reported similar findings, demonstrating a reduction in vasopressors utilized prior to organ recovery when methylprednisolone or hydrocortisone was administered to the donor. However a randomized clinical trial by Venkateswaran et al. [38] concluded, as a secondary outcome, that methylprednisolone did not improve cardiovascular function. A previous work by Venkateswaran et al. [9] evaluated the effects of 1 g of methylprednisolone administration to brain-dead organ donors 7 hours prior to lung explantation, demonstrating no effect on increasing lung yields but a reduction in lung water accumulation.

Despite this complex and contradictory background there are physiological reasons to think that steroids can improve hemodynamics. Corticoids as prednisolone have a mineralocorticoid effect which increase the sodium reuptake in the kidney [39]. Moreover corticoids could increase the endogenous production of adrenaline. These effects increase blood pressure and it could be one reason of the hemodynamic improvement in our model [40].

Another reason for a better hemodynamic performance could be the beneficial effect of prednisolone in microcirculation. Sack et al. [41] show in an animal model that prednisolone prevents injury of the bowel induced by extracorporeal circulation using* in vivo* microscopy based on labeled dextran. The influence of brain death over microcirculation has been studied by Simas et al. [42] proving that mesenteric microcirculation is decreased during brain death measured with* in vivo* microscopy. Yamagami et al. [43] using a similar methodological approach proved that hepatic microcirculation is affected by brain death. However more research needs to be performed to understand the impact of microcirculation impairment during brain death and its potential treatment.

To delineate the anti-inflammatory effect of prednisolone we tested three different doses of this drug after brain death induction in our animal model. We found a strong anti-inflammatory response due to prednisolone treatment, measured by IL-6 plasma levels and the expression of* IL-6, IL-1β*, and* MCP-1* in liver and kidney tissue. These results are in line with a randomized clinical trial by Kotsch et al. [6] demonstrating a significant downregulation of proinflammatory cytokines which was associated with a reduced incidence of acute rejection in the liver transplantation setting. In contrast, Amarschek et al. [44] conclude that steroid donor treatment did not improve outcomes after liver transplantation, but no inflammatory markers were measured in this study. Others in the realms of kidney transplantation have failed to detect a significant improvement in the incidence or duration of posttransplantation acute renal failure in allograft recipients when 1 g of methylprednisolone is administered to donors 3 hours prior to explantation, despite the suppression of inflammatory response in transplanted kidneys [45].

In a previous study we showed that prednisolone administered before the induction of brain death in fact reduced inflammation measured by IL-6 plasma levels and renal and liver expression of proinflammatory cytokines like* IL-6, IL-1β, MCP-1*, and* TNF-α*. Interestingly prednisolone pretreatment did improve renal function measured by creatinine plasma levels but did not improve liver cellular injury measured by AST, ALT, and LDH plasma levels [31]. We think that this differing effect may be related with the persistence of complement activation (*C3*) in the liver and downregulation of protective cellular mechanisms such as heat shock proteins.

Thus, contrary to our hypothesis and our other study, post-brain death induction therapy with prednisolone did not improve organ quality in terms of renal function, as measured by creatinine levels. Additionally and in contrast to our previous results, we found a persistent complement activation in the kidney of BD rats treated with prednisolone treatment. One of the differences which may account for this, in part, is the different durations between time of administration and time of retrieval of organs. In the current study prednisolone was administered after an hour of BD and BD was maintained for 3 hours. This time window may have been too short to observe the benefits seen in the previous study.

In addition, the relative expression of genes related with cellular protection (*HSP-70* and* HO-1*) was not modified by prednisolone treatment. In addition, PMN influx was not reduced in the liver nor kidney by prednisolone treatment. This may reflect the ongoing cellular injury in both organs and the short follow-up period after prednisolone administration.

In previous studies we have shown that following the onset of BD apoptosis is increased in both the liver and kidney [27, 31]. However, apoptosis seems to be triggered independently of the systemic inflammatory response, perhaps by other factors such as cellular stress, hypoxia, and altered metabolism. The complement system can be activated by apoptotic cells. Indeed, the pattern recognition molecule (PRM) C1q activates complement by recognizing distinct structures directly on microbial and apoptotic cells. The complement system creates cellular injury by promoting phagocytosis and anaphylaxis reaction [46]. Therefore, we hypothesize that the persistent* C3* complement expression may account for the lack of responsiveness of the kidney and liver to the anti-inflammatory effects of prednisolone. Further experiments need to be performed to test this hypothesis.

However, work performed by Damman et al. [47] supports this idea, as they have demonstrated that BD induced complement activation and cell injury can be targeted and improves the outcomes of transplantation. Considering these results, maybe the combination of steroids and anticomplement activation therapy could be a better approach to improve organ quality in BD donors.

## 5. Conclusion

Our study has demonstrated that prednisolone administration following brain death improves the hemodynamic stability of a brain-dead organ donor. Despite a clear effect on downregulating systemic proinflammatory cytokines, we did not detect a significant improvement in surrogate markers for organ function.

## Figures and Tables

**Table 1:** Primer sequences used for Real-Time PCR.

Gene	Primers	Amplicon size (bp)
*TNF-α*	5′-GGCTGCCTTGGTTCAGATGT-3′	79
	5′-CAGGTGGGAGCAACCTACAGTT-3′	

*IL-1β*	5′-CAGCAATGGTCGGGACATAGTT-3′	75
	5′-GCATTAGGAATAGTGCAGCCATCT-3′	

*IL-6 *	5′-CCAACTTCCAATGCTCTCCTAATG-3′	89
	5′-TTCAAGTGCTTTCAAGAGTTGGAT-3′	

*C3 *	5′-CAGCCTGAATGAACGACTAGACA-3′	96
	5′-TCAAAATCATCCGACAGCTCTATC-3′	

*MCP-1 *	5′-CTTTGAATGTGAACTTGACCCATAA-3′	78
	5′-ACAGAAGTGCTTGAGGTGGTTGT-3′	

*HO-1 *	5′-CTCGCATGAACACTCTGGAGAT-3′	74
	5′-GCAGGAAGGCGGTCTTAGC-3′	

*HSP-70 *	5′-GGTTGCATGTTCTTTGCGTTTA-3′	80
	5′-GGTGGCAGTGCTGAGGTGTT-3′	

*BAX *	5′-GCGTGGTTGCCCTCTTCTAC-3′	74
	5′-TGATCAGCTCGGGCACTTTAGT-3′	

*Bcl-2 *	5′-CTGGGATGCCTTTGTGGAA-3′	70
	5′-TCAGAGACAGCCAGGAGAAATCA-3′	

**Table 2:** HAES and noradrenaline requirements.

Group	Total HAES 10% infusion (mL)	Total noradrenaline infusion (mL)
Saline	2.8 ± 1.9	2.3 ± 2.2
22.5 mg/Kg prednisolone	1.6 ± 1.3	0.3 ± 0.8
12.5 mg/Kg prednisolone	1.5 ± 0.8	0.2 ± 0.5
5 mg/Kg prednisolone	1.7 ± 1.1	0.3 ± 0.4
